# Factors contributing to learning satisfaction with blended learning teaching mode among higher education students in China

**DOI:** 10.3389/fpsyg.2023.1193675

**Published:** 2023-06-29

**Authors:** Xiaoliang Cheng, Weilan Mo, Yujing Duan

**Affiliations:** ^1^Education Evaluation and Supervision Division, Chengdu University of Technology, Chengdu, China; ^2^Student Office, Chengdu University of Traditional Chinese Medicine, Chengdu, China

**Keywords:** blended teaching, student satisfaction, affecting elements, Chinese university, variance analysis

## Abstract

Blended learning has increasingly grown in importance as a method of classroom instruction in Chinese higher education classrooms in the context of fast-evolving network information technology, higher standards of educational informatization, and growing attention to the reform of teaching modes in higher education. The efficiency of blended learning can be increased by better understanding the students’ learning satisfaction and its key influencing factors. Based on the theories of constructivism and phenomenology, the study constructs an index system of student satisfaction with blended learning in higher education, and conducts a questionnaire survey on 650 students with blended learning experience in 6 universities in Sichuan Province, China, obtaining 598 valid questionnaires after reviewing the collected questionnaires for missing values. This study uses descriptive statistical analysis (DSA), one-way ANOVA, Pearson correlation analysis, and multiple linear regression (MLR) to analyze the effects of each factor in the index system on satisfaction. Results indicate that the overall level of student satisfaction with blended learning in universities is moderately high, with students’ self-satisfaction being the lowest, and that substantial disparities exist in the evaluation of satisfaction with blended learning on various online resources, online teaching forms, and offline teaching methods. This study applies multiple linear regression (MLR) to conclude that students’ learning attitudes, curriculum design, and teachers’ teaching methods are the most important factors influencing satisfaction with blended learning in universities. Results indicate that a blended learning system be built from the three dimensions of students, teachers, and curriculum, offering a theoretical foundation and point of reference for the ongoing reform of blended learning in higher education. The study is of great significance in optimizing teaching quality and deepening the reform of blended learning.

## Introduction

With the rapid expansion of information, technology, and the Internet in the 21st century, the relationship between higher education and information technology has grown closer (Mitchell and Forer, 2010). The development and reform of higher education have gradually moved in the new direction of curriculum and IT integration (Gerbica, 2011; Xu and Shi, 2018). Blended learning is part of the ongoing convergence of two archetypal learning environments (Ates, 2009). The “Implementation Opinions of the Ministry of Education on the Construction of First-class Undergraduate Curriculum” issued by the Ministry of Education of the People’s Republic of China (2019), clarified that online and offline blended courses should be customized to local, school, and course-specific circumstances, placing special emphasis on the reform and innovation of curriculum content and teaching methods. Blended learning has made a historic leap in recent years in China. Taking Chinese MOOCs (Massive Open Online Courses) as an example, universities in western China implemented blended learning for 1.26 million courses in 2022 alone, with 210 million students participating in it, significantly raising the teaching quality of teachers and the learning outcomes of students in this region (People’s Daily, 2023). “The new integration of technology and education will unleash a new revolution, and blended learning will become the new normal of higher education in the future” suggested Wu Yan (Xinhuanet, 2021), director of the Department of Higher Education of the Ministry of Education. Due to its dynamic and modern nature, blended learning brings higher education a fresh lease on life.

Blended learning undoubtedly demonstrates a new route for future teaching development in the era of ever-changing network information technology (Gerbica, 2011), high standards of educational informatization (Picciano and Dziuban, 2007), and growing attention to teaching mode reform (Garrison and Kanuka, 2004). China’s blended learning combines the benefits of traditional and online education to achieve the dual mode of “online + offline,” and the introduction of this teaching mode will lead to significant advancements in higher education (Zhu and Hu, 2021). Blended learning is currently being popularized and developed in both domestic and international higher education (Garrison and Kanuka, 2004). Increasing attention is also being given to modeling the effectiveness of e-learning mechanisms, including factors such as the use of online assessments, students’ attitudes toward e-learning mechanisms, and the application of different teaching methods (Carver et al., 2004; Chang and Tung, 2008; Sivo et al., 2010; Sadeghi et al., 2014). In-depth investigation and research on students’ satisfaction with blended learning, as well as further evaluation of various influencing factors involved in blended learning, play a vital role in enhancing blended learning theory and practice.

This study starts from the research results on student satisfaction with blended learning in higher education, both domestically and internationally. Firstly, it comprises the basic concepts and related theories of blended learning, student satisfaction, constructivist learning theory, etc. Secondly, through two modes of online and offline questionnaires, a survey was conducted on 598 students of different majors and grades in six universities in China to examine the implementation of blended learning. Thirdly, descriptive statistical analysis (DSA), one-way ANOVA, Pearson’s correlation analysis, and multiple linear regression (MLR) were used to analyze the influence of each factor in the index system on satisfaction. It analyzes the current situation and problems of university students’ satisfaction with blended learning from students’ perspectives and explores the factors that affect satisfaction with blended learning in higher education. The study further explores the focus and direction of blended learning improvement, which provides a reference for the reform and innovation of blended learning and is of great significance in optimizing teaching quality and deepening the reform of blended learning.

## Research review

### Research on blended learning

Domestic and international scholars have undergone a metamorphosis with the continuous development of blended learning, moving from basic conceptual research to technology application research, as well as evaluation and management research. Studies on the effects of blended learning and its influencing factors are becoming more in-depth.

Garrison and Vaughan (2008) indicated that blended learning was developed from the strengths of face-to-face and distance learning. According to Linder (2017), the blended learning method is a way of using technology to give students access to a variety of learning environments, where face-to-face activities are often combined with technology-mediated activities to allow students to have more active learning opportunities and more targeted instruction when learning outside of the classroom. Pape (2010) proposed that the advantages of blended learning include providing students with multiple ways to demonstrate their knowledge while engaging various learning styles and helping students build independent and self-directed learning skills, which are essential for lifelong learners. Tayebinik and Puteh (2013) pointed out that blended learning promotes a stronger sense of engagement and community than traditional face-to-face or fully online teaching blended learning is a flexible, scalable, and meaningful way of teaching and learning.

Blended learning was initially brought to China in 2003 by Zhu and Meng (2003), who suggested that it can be defined as instruction applying multiple delivery techniques in order to optimize learning outcomes and the cost of learning program delivery. Based on the level of teaching space, Chen et al. (2019) proposed that blended learning expands students’ learning beyond the typical classroom’s dozens of square meters and out into the online and offline worlds, and is a mixture of learning environments as well as learning spaces. Wei and Tan (2021) believes that blended learning is a teaching mode guided by behaviorist and constructivist learning theories and with the aid of modern educational technology, Internet technology, and other technical means. It deeply integrates traditional face-to-face instruction, practical instruction, and online instruction to achieve the highest teaching efficiency and effect. In general, blended learning involves the use of different teaching resources, “online + offline” multiple learning platforms as the carrier, more thorough learning objectives as the guide, flexible use of a variety of teaching methods and organizational forms, fully combining the benefits of online and offline classrooms.

### Research on student satisfaction

The concept of student satisfaction, based on customer satisfaction, was formally introduced into the field of education at the end of the last century by local and international scholars. The higher education sector has become increasingly aware that students’ expectations and needs are similar to those of a service business, and has paid further attention to fulfilling their expectations and needs (Cheng and Tam, 1997). The learning experience and satisfaction of students, who are the subjects of education, are important criteria for evaluating teaching quality (Guolla, and Michael., 1999).

Several academics have various definitions of the concept of learning satisfaction based on different study viewpoints. One is the idea of discrepancy based on a comparison of students’ learning before and after. It is the psychological satisfaction or dissatisfaction of students’ perception of the service level of teaching during the entire teaching service, compared with their self-expectations or demands before learning. The second is the learning experience and learning outcomes perceived by the students themselves (Zhu et al., 2020), and the concept of student satisfaction is proposed in terms of the students’ own holistic nature, which is the student’s subjective evaluation of various outcomes and experiences related to education (Oliver, 1997). The third is to propose the concept of student satisfaction with particular aspects during school, also referred to as the elemental concept. The idea places a focus on satisfaction at specific levels, including educational methods, living services, hardware facilities, and so on. As a development and improvement of the holistic idea, it also incorporates other elements such as perception of interaction, relationship, support for learning and development, and perception of gain (Liu and Fan, 1995).

With regard to student satisfaction, there is an overwhelming body of research that demonstrates that students have greater satisfaction with blended courses, compared with both traditional face-to-face and fully online modes of education (Owston et al., 2006; Collopy and Arnold, 2009; Castle and McGuire, 2010; Farley et al., 2011). Research on the influencing factors of blended learning satisfaction shows that having continuous access to the instructor is perceived as an important factor in students’ satisfaction with blended learning (Martinez-Caro and Campuzano-Bolarin, 2011). Some students report that they receive instructor feedback and their grades faster than in traditional courses (Korr et al., 2012). Taghizadeh and Hajhosseini (2021) investigated 140 TEFL (Teaching English as a Foreign Language) students’ attitudes, interactions, and relationships with blended learning quality. They found that students were most satisfied with teacher-student interaction in blended learning, and had the largest impact coefficient on teaching quality and satisfaction. Among them, teachers’ teaching ability contributed significantly more to satisfaction than teachers’ teaching attitude. Wu Jen-Her through confirmatory factor analysis (CFA), Tennyson Robert D, and Hsia Tzyh-Lih (Wu et al., 2010) concluded that self-efficacy, performance expectations, system functions, content characteristics, interaction, and learning atmosphere are the main factors that affect students’ learning satisfaction with BELS (Blended E-Learning System) in blended learning. While learning atmosphere and performance expectations significantly affect learning satisfaction, teacher-student interaction significantly affects learning atmosphere, and self-efficacy, system function, content characteristics, and interaction significantly affect performance expectations.

### Research on constructivist learning theory

Constructivist learning theory first originated from the Swiss educationalist Piaget’s theory of children’s cognitive development and is now commonly considered to comprise Piaget’s individual constructivism and Vygotsky’s social constructivism. Gordon (2008), Neo and Neo (2009), and Felder (2012) hold the opinion that constructivism has emerged as a powerful theory for explaining how humans learn about the world around them and how new knowledge is formed. According to this theory, the formation of the learner’s own knowledge and the development of cognitive structures do not result from the educator’s face-to-face instruction, but rather from the interaction of the learner with the surrounding environment, learners constructing their own cognitive structures through both assimilation and conformity modes of behavior, and therefore it is fundamentally personal and depends on the learning environment, knowledgeable and experienced teachers, and experiences in social interactions (Grabinger and Dunlap, 1995). The process of constructing cognitive structures is a form of active absorption of knowledge by the learner, instead of being passive, emphasizing the subjectivity of the learner (Gordon, 2008). Constructivist learning theory indicates that the learning environment plays a crucial role for learners as they continually build and update their own knowledge systems. Individuals who want to better build a learning system in line with their own are to a certain extent influenced by the learning environment, and “scenario,” “cooperation,” “conversation,” and “meaning construction” are the four major factors of the learning environment.

The teacher’s unilateral transfer of knowledge to students in traditional instruction is transformed in blended learning, as students shift from passive receivers to active constructors through the continual interplay of their existing and new knowledge. By providing rich and diversified resources, blended learning creates attractive learning scenarios that students find appealing, encourages their participation and initiative in communication and interaction, piques their interest in learning, and makes them active participants in their education.

### Research on the complex adaptive blended learning system

The Complex Adaptive Blended Learning System first arose in the disciplines of physics, mathematics, and chemistry to construct a framework for complex adaptive systems, which was intended to be used to enhance knowledge of dynamic and complex topics and nonlinear systems such as neural, ecological, galactic, and social systems (Hadzikadic et al., 2010). Complex adaptive systems are described as living open systems that “exchange matter, energy, or information across boundaries and use this energy exchange to maintain their structure” (Cleveland, 1994).

To fill a gap in blended learning research and further enhance the systematic understanding of blended learning research and practice, Wang et al. (2015) call for a systematic analysis of blended learning (BL) programs by adopting a framework called the Complex Adaptive Blended Learning System (CABLS), which, they argue, “helps to reveal the untapped potential and key issues … such as the provision of learning support, the promotion of institutional engagement, and the non-linear relationships among subsystems in blended learning.” Six learning elements are therefore proposed through the Complex Adaptive Blended Learning System (CABLS): Learner, Teacher, Technology, Content, Learning Support, Institution, each with its own characteristics and subsystems (Cleveland-Innes and Wilton, 2018).

These six subsystems will interact in a nonlinear and dynamic manner, consistent with other complex adaptive systems. “Its own characteristics and internal driving forces, depending on surrounding subsystems, to maintain its vitality” (Wang et al., 2015). At the same time, each of these subsystems has its own characteristics or features that are self-motivating while interdependent to maintain competitiveness, and they will interact with each other to form a blended learning system. The CABLS framework aims to “promote a deeper and more accurate understanding of the dynamics and adaptability of hybrid learning” (Wang et al., 2015).

Using this framework, Wang et al. (2015) reviewed a 20-month period (January 2013 to August 2014) and found that research on BL was scattered over that time period, with 95% of the review articles focused on learners, followed by content (79%) and technology (54%). This percentage drops sharply when it comes to focusing on teachers (32%), institutions (17%), and learning support (15%), and there is no systematic study of the links between these elements since most studies only examine one element. Therefore, there is a need to focus on the interconnections between elements and to conduct a more in-depth study of the BL situation.

In summary, the Complex Adaptive Blended Learning System proposes that a set of logically interrelated and hierarchically different systems can be formed through effective classification of six learning elements. The resulting classification system generally has three characteristics: first, the different categories are logical; second, the research results are consistent with previous research results; and third, the classification results are as complete as possible and can cover different categories. According to the topic and specificity of this study, therefore, this study analyzes and organizes the relevant influencing factors in three dimensions: learning dimension, teaching dimension, and curriculum dimension. The learning dimension covers learning attitudes and learning behaviors. The teaching dimension covers teaching attitudes, teaching methods, and teaching abilities. The curriculum dimension covers curriculum design and platform design. These three dimensions cover the six subsystems of the Complex Adaptive Blended Learning System that are more likely to facilitate “deep learning,” and the research findings can assist us in making better decisions when implementing blended learning, and effectively support our learners in achieving deeper and more meaningful learning.

## Methods and investigations

### Data collection and sample description

A questionnaire survey was conducted between September 2021 and January 2022 among 650 college students with blended learning experiences in six universities in Sichuan Province, China, through online and paper questionnaires to ensure the accuracy of the data sample. A total of 650 questionnaires were distributed, and 628 questionnaires were collected, with a return rate of 97%. After checking the missing values of the questionnaires, it was found that a total of 30 questionnaires were missing incomplete. Therefore, 598 valid questionnaires were obtained, with an effective rate of 95%. Among the 598 samples, in terms of gender ratio, 325 female students accounted for 54.3% of the total and 273 male students accounted for 45.7%, with a larger proportion of female students. In terms of grade ratio, 206 freshmen accounted for 34.4%, 147 sophomores accounted for 24.6%, 125 juniors accounted for 20.9%, and 120 seniors accounted for 20.1%, with a large proportion of freshmen, but the overall distribution was balanced. In terms of major categories, humanities and social sciences, science and technology, agriculture and medicine, and arts and sports accounted for 33.9%, 33.3%, and 32.8%, respectively. The distribution of students’ major categories in the survey is more balanced. In terms of politic countenance, the total number of students who are members of the Communist Youth League is 472, accounting for 78.9%, which is significantly higher than that of members of the Communist Party of China (including probationary Party members) and the masses.

### Questionnaire design

This study is based on a questionnaire survey to quantify the indicators and obtain relevant data. The questionnaire was designed in a targeted and quantitative way by consulting relevant books and materials, and combining them with the actual situation of blended learning in universities. The scale index design of this study is divided into the following two stages: first, after discussing with experts and teachers responsible for the construction of blended learning courses in the university, as well as combining the opinions and suggestions of students who have blended learning experiences, continuous modifications are made in terms of suitability, classification, and semantics, and so on; Secondly, a pre-survey was conducted to distribute questionnaires in a certain range, and the content of the questionnaires was analyzed using the high-low grouping method to ensure that the significance level of each indicator was less than 0.05. Finally, a total of 31 questions were used to construct the “College Blended Learning Student Satisfaction Scale” from 12 indicators, which comprise learning platform, online teaching resources, online teaching forms, offline teaching methods, students’ learning attitudes and learning behaviors during learning, teachers’ teaching attitudes, teaching methods, and teaching abilities, curriculum design and platform design, and teaching satisfaction, and so on. The scale is divided into three parts: the first part is personal information, which includes gender, grade, major category, and politic countenance; the second part is a survey on the current situation of blended courses, which aims to investigate whether there is a significant distinction between various teaching processes on students’ satisfaction; the third and fourth parts are the main questionnaire (see Table 1), which includes learning dimension, teaching dimension, curriculum dimension and satisfaction of teaching, and the variables are all scored on a five-point Likert scale, corresponding to scores from one to five, and respondents were able to choose the most appropriate answer for their actual situation.

**Table 1:** Questionnaire structure of student satisfaction and influencing factors of blended learning in universities.

Dimension		Indicators		Corresponding question number
Part I	Personal information	Gender, grade, major category, and politic countenance		A1–A4
Part II	Current situation of blended courses	Learning platform		B1
		Online teaching resources		B2
		Online teaching format		B3
		Offline teaching method		B4
Part III	Learning dimension	Learning attitude	Learning interest	C1
			Learning awareness	C2
			Learning self-efficacy	C3
		Learning behavior	Learning concentration	C4
			Learning reflection	C5
			Level of communication and interaction	C6
	Teaching dimension	Teaching attitude	Careful preparation before and after class	C7
			Timely feedback	C8
		Teaching method	Active classroom interaction	C9
			Rich teaching methods	C10
		Teaching ability	Information technology capability	C11
			Teaching and research capabilities	C12
	Curriculum dimension	Course design	Richness of course content	C13
			Reasonableness of course objectives	C14
			Appropriateness of online and offline distribution	C15
			The scientific nature of the assessment	C16
		Platform design	Ease of platform operation	C17
			Completeness of platform functions	C18
Part IV	Satisfaction of teaching			D1–D5

### Exploratory factor analysis and validity testing

The scales in the third and fourth parts were not designed as classical scales, wherein the principal component analysis of exploratory factors was used to verify the correspondence between the items and the variables. When KMO (Kaiser-Meyer-Olkin) > 0.9, the effect of factor analysis is ideal (Wu, 2003). According to the test results of different question items, the total KMO value of the questionnaire is 0.974 > 0.9, the Sig value is 0.00 < 0.05, the cumulative explained variance is 79.73%, and the variance explained after rotation of each factor was above 10%, indicating that the exploratory factor analysis results are good. The factor loading coefficients of each factor are higher than 0.6, which indicates good overall scale validity (Table 2).

**Table 2:** Questionnaire validity and cumulative explained variance analysis.

Cumulative Explained Variance		79.730%
Kaiser-Meyer-Olkin measure of sampling adequacy		0.974
Bartlett’s Sphericity test	Chi-square approximation	20410.570
	df	325
	Sig.	0.000

This study adopts Cronbach’s alpha reliability analysis. The Cronbach’s alpha of the three dimensions of students, teachers, and curriculum in the third part are 0.945, 0.976, and 0.969 respectively, and the Cronbach’s alpha of teaching satisfaction in the fourth part is 0.975, the alpha coefficient of each dimension was above 0.9. The α coefficients of each dimension are above 0.9, which shows that the questionnaire has good reliability (Table 3).

**Table 3:** Questionnaire validity analysis.

Indicators		Cronbach’s alpha	Number of projects
Part III	The credibility of learning dimension	0.945	6
	The credibility of teaching dimension	0.976	6
	The credibility of curriculum dimension	0.969	6
Part IV	The credibility of satisfaction of teaching	0.975	5
Overall Credibility of the Questionnaire		0.985	23

## Results and date analysis

### Analysis of students’ satisfaction with blended learning in universities

The survey data is shown in Table 4, from which the average value of satisfaction with blended learning is 3.49, which is at the upper-middle level. Among them, more than 60% of the students agreed or strongly agreed with three indicators, which shows that satisfaction with blended learning in universities is good. The students who agree and strongly agree with the indicator “In general, I am satisfied with the blended learning.” are the most, reaching 61.7%, and the students who agree and strongly agree with the indicator “By studying this course, I have completed the expected learning objectives or tasks.” are the least, only 50.8%, indicating that nearly half of the students are dissatisfied with the learning objectives and completion of learning tasks after the course.

**Table 4:** Satisfaction rate of blended learning in universities (%).

Indicator	Strongly disagreed	Disagree	Neutral	Agree	Strongly agree	Mean value	Population mean	Standard deviation
In general, I am satisfied with the blended learning.	2.0	13.5	22.7	43.3	18.4	3.63	3.49	0.855
If possible, I will choose blended learning for learning.	1.8	13.4	24.6	44.1	16.1	3.59		
I would recommend this blended course and its instructor to others.	1.8	16.6	20.2	40.5	20.9	3.61		
Compared with traditional learning and online learning alone, blended learning g has more advantages.	2.0	26.1	21.6	40.0	10.4	3.35		
By studying this course, I have completed the expected learning objectives or tasks.	1.7	22.6	25.9	43.3	7.5	3.31		

### Satisfaction analysis based on three dimensions of teaching, learning, and curriculum

Table 5 displays the results of the satisfaction survey for the three dimensions of teaching, learning, and curriculum, and college students are the least satisfied with their own learning status. Tables 6–8 display the satisfaction rates for each indicator of the three dimensions. There are obvious differences in their satisfaction with self-attitude and self-behavior, which reflect the disunity of knowing and doing. The mean value of students’ satisfaction with teachers’ teaching attitude is greater than the mean value of satisfaction with teaching methods and teaching ability, which indicates that teachers’ teaching techniques and abilities need to be further optimized. In terms of curriculum, the design of teaching contents, the hours and the frequency of instruction fall short of properly fulfilling the needs of the students.

**Table 5:** Satisfaction statistics of the three dimensions of teaching, learning, and curriculum in blended learning in universities.

	Index mean	Index standard deviation	Population mean	Standard deviation
Learning attitude	3.38	0.956	3.36	0.819
Learning behavior	3.35	0.866		
Teaching attitude	3.66	0.989	3.62	0.843
Teaching method	3.61	0.889		
Teaching ability	3.61	0.927		
Course design	3.56	0.861	3.59	0.852
Platform design	3.62	0.927		

**Table 6:** Satisfaction rate of each indicator in learning dimensions of blended learning in universities (%).

Indicator		Strongly disagreed	Disagree	Neutral	Agree	Strongly agree	Mean value
Learning attitude	I have some interest in blended learning courses.	7.7	16.9	34.8	24.6	16.1	3.24
	This course is very important for my future development.	2.7	13.4	28.6	35.1	20.2	3.57
	Before the course, I will set learning goals and be confident that I will be able to complete this course.	2.8	23.2	22.9	39.1	11.9	3.34
Learning behavior	I am more focused in online or offline courses alone.	4.2	13.7	36.5	28.9	16.7	3.40
	I often reflect on my studies in my spare time, judging my mastery of the course.	3.3	16.9	38.5	31.8	9.5	3.27
	During blended learning, I will have active communication and interactions with teachers and classmates in various ways.	2.7	15.6	34.4	35.1	12.2	3.38

**Table 7:** Satisfaction rate of each indicator in teaching dimensions of blended learning in universities (%).

Indicator		Strongly disagreed	Disagree	Neutral	Agree	Strongly agree	Mean value
Teaching attitude	In blended learning, teachers carefully prepare lessons before class and are responsible after class.	1.8	16.6	17.9	41.0	22.7	3.66
	Timely feedback on questions, discussions and assignments are provided by the teacher during blended learning.	2.7	13.7	19.6	42.6	21.4	3.66
Teaching method	Teachers frequently develop interactive environments and interactive scenarios during blended learning.	3.0	13.0	22.4	41.5	20.1	3.63
	In blended learning, teachers adopt various teaching methods such as lecture, case study, discussion and practice.	2.0	14.9	22.4	41.5	19.2	3.61
Teaching ability	Teachers integrate information technology deeply with the subjects in blended learning, and have good IT skills.	2.2	18.1	22.7	39.8	17.2	3.52
	In blended learning, the teachers are knowledgeable and have good teaching ability.	1.5	10.5	20.2	41.5	21.3	3.70

**Table 8:** Satisfaction rate of each indicator in curriculum dimensions of blended learning in universities (%).

Indicator		Strongly disagreed	Disagree	Neutral	Agree	Strongly agree	Mean value
Course design	The content and resources of both online and offline learning are rich and interesting, and can satisfy my desire to learn.	2.2	12.2	31.6	40.6	13.4	3.51
	The objectives of both online and offline learning are well designed	1.5	12.0	25.8	44.0	16.7	3.62
	Appropriate design of both online and offline learning (including the hours, frequency, interaction and connection of online and offline learning, etc.).	1.3	10.4	33.3	41.0	14.0	3.55
	The evaluation and assessment of the course is designed in a scientific way.	1.0	15.6	26.9	40.3	16.2	3.56
Platform design	Great convenience and ease of learning with the learning platform.	1.2	12.2	23.9	41.0	21.7	3.70
	The various functions of the learning platform (such as notes, tests and interactions, etc.) are complete, and I can make good use of them	1.8	11.9	24.1	41.8	17.4	3.64

### Differential analysis of students’ satisfaction with blended learning in universities

The analysis of the variability of satisfaction based on individual characteristics of college students by independent sample *t*-test and one-way ANOVA test results is shown in Table 9. The significant *p*-value is greater than 0.05, while the results show that no significant difference exists between the satisfaction of different personality characteristics.

**Table 9:** Satisfaction statistics of college students with different characteristics on blended learning.

Characteristics	Category	*N*	Mean value	Standard deviation	T-value	F-value
Gender	Male	273	3.44	0.891	−1.238	
	Female	325	3.53	0.824		
Grade	Freshman	206	3.35	0.921		1.841
	Sophomore	147	3.36	0.789		
	Junior	125	3.52	0.708		
	Senior	120	3.79	0.885		
Major	Humanities and Social Science	203	3.52	0.839		1.464
	Science, Engineering, Agriculture and Medicine	199	3.50	0.860		
	Arts and Sports	196	3.44	0.869		
Politic countenance	Chinese Communist Party members (including probationary Party member)	54	3.62	0.806		1.384
	Communist Youth League member	472	3.48	0.880		
	The masses	72	3.38	0.709		

^*^p < 0.05, ^***^p ≤ 0.001.

One-way ANOVA were conducted on the satisfaction of teaching on different learning platforms, different online resources, different online teaching forms, and different offline teaching methods, and the details are shown in Tables 10–13. The results show that there is no significant difference in students’ satisfaction with blended learning depending on the learning platform. However, a significant difference exists in students’ satisfaction with blended learning due to different online resources, online teaching forms, and offline teaching methods. Teacher-built fragmented courseware and teacher-built systematic courses, online teaching forms with fixed locations and time, and mutual discussions and student-led lectures are more popular among students. Adding technology to in-person teaching and learning may foster engagement and improve learning outcomes. According to the SAMR (Substitution, Augmentation, Modification, Redefinition) model, blended learning adds technology to the online and offline classroom and is technology-supported learning. In general, blended learning in universities basically covers substitution, augmentation, and modification, and less for redefinition. In the comparative analysis, significant differences were found in students’ satisfaction with blended instruction based on different offline teaching methods. Mutual discussion and student-led lectures were more popular among students. These two teaching methods have brought each other closer, improved students’ interaction, communication, and engagement, given students more initiative, and made it easier for teachers to monitor students’ learning progress and status. There were significant differences in student satisfaction with blended learning based on different online teaching forms. The mean value of student satisfaction with blended learning was highest among fixed-location and fixed-time modes of online teaching forms. The main distinction between synchronous and asynchronous learning is whether the time and location of learning are consistent, with synchronous learning guaranteeing students’ learning pace and efficiency and asynchronous learning testing students’ self-motivation and autonomy. There is a problem with online learning integrity due to the irresponsible behavior of some students toward learning. Students simply play learning videos without carefully watching them, affecting their learning effectiveness, which goes against the original purpose of online learning.

**Table 10:** Statistics of students’ satisfaction with blended learning on different learning platforms.

Platform Type		*N*	Mean value	Standard deviation	F-value
Super star learning APP	Not selected	83	3.32	0.946	1.633
	Select	515	3.51	0.838	
MOOC and client ware	Not selected	280	3.40	0.894	1.627
	Selected	318	3.64	0.754	
QQ group and WeChat group	Not selected	394	3.41	0.871	1.727
	Selected	204	3.55	0.838	
Ding talk	Not selected	481	3.47	0.865	1.092
	Selected	117	3.56	0.812	
Other	Not selected	534	3.48	0.834	1.822
	Selected	64	3.55	1.004	

^*^p < 0.05, ^***^p ≤ 0.001.

**Table 11:** Statistics of students’ satisfaction with blended learning of different online resources.

Online resource type		*N*	Mean value	Standard deviation	F-value
Teacher-built systematic courses	Not selected	150	3.34	0.745	5.734^*^
	Selected	448	3.72	0.846	
National-level open course	Not selected	346	3.50	0.886	5.423^*^
	Selected	252	3.62	0.735	
Other courses	Not selected	397	3.45	0.813	5.645^*^
	Selected	201	3.57	0.886	
Teacher-built fragmented courseware	Not selected	401	3.56	0.834	6.547^*^
	Selected	197	3.87	0.847	

^*^p < 0.05, ^***^p ≤ 0.001.

**Table 12:** Statistics of students’ satisfaction with blende learning in different online teaching forms.

Online teaching format		*N*	Mean value	Standard deviation	F-value
Unfixed locations, unfixed time	Not selected	145	3.67	0.953	6.334^*^
	Selected	453	3.44	0.868	
Unfixed locations, fixed time	Not selected	361	3.45	0.845	6.867^*^
	Selected	237	3.52	0.795	
Fixed locations fixed time	Not selected	556	3.45	0.865	5.235^*^
	Selected	42	3.78	0.878	

^*^p < 0.05, ^***^p ≤ 0.001.

**Table 13:** Statistics of students’ satisfaction with blended learning of different offline teaching methods.

Offline teaching method		*N*	Mean value	Standard deviation	F-value
Teacher-led lectures	Not selected	120	3.79	0.657	13.987^***^
	Selected	478	3.23	0.889	
Mutual discussions	Not selected	278	3.53	0.788	9.6044^***^
	Selected	320	3.67	0.722	
Student-led lectures	Not selected	302	3.49	0.546	10.4554^***^
	Selected	296	3.25	0.912	

^*^p < 0.05, ^***^p ≤ 0.001.

## Analysis and discussion

### Correlation analysis of influencing factors

To explore the correlation between student satisfaction with the three factors of the learning dimension, teaching dimension, and curriculum dimension of blended learning in universities, this study processed the data of the three-dimensional scales accordingly through Pearson correlation analysis and multiple linear regression (MLR) to ensure that the factors affecting satisfaction with blended learning in universities could be reflected. Pearson correlation analysis was conducted between the three influencing dimensions (learning dimension, teaching dimension, and curriculum dimension) and students’ satisfaction, and the results are shown in Table 14. According to the results of correlation analysis, the *p*-values corresponding to the satisfaction of blended learning and the three factors of learning dimension, teaching dimension, and curriculum dimension are all close to 0, indicating that the learning dimension, teaching dimension, and curriculum dimension are significantly positively correlated with students’ satisfaction with blended learning, i.e., the increase in student satisfaction with themselves, teachers, and curriculum would be followed by an increase in satisfaction with blended learning. The correlation coefficients are less than 0.70, so there is no multicollinearity phenomenon. The correlation coefficients of learning, teaching, and curriculum with students’ satisfaction were 0.457, 0.331, and 0.414, respectively, which shows that the correlation between the learning dimension and students’ satisfaction is the strongest, followed by the curriculum dimension, and the teaching dimension is the weakest.

**Table 14:** Correlation Matrix between influencing factors and satisfaction of blended learning.

	Satisfaction of blended learning	Learning dimension	Teaching dimension	Curriculum dimension
Satisfaction of blended learning	1			
Learning dimension	0.457^**^	1		
Teaching dimension	0.331^**^	0.458^**^	1	
Curriculum dimension	0.414^**^	0.411^**^	0.320^**^	1

^**^ Significant at the 0.01 level.

### Regression analysis of influencing factors

The correlation analysis yields significant correlations among the variables. The next step of regression analysis is carried out. The multiple linear regression (MLR) analysis method is adopted to verify whether a significant correlation exists between the learning dimension, the teaching dimension, the curriculum dimension at all levels, and the satisfaction of teaching, and then to understand the impact of each dimension on the satisfaction with blended learning. The learning dimension includes learning attitude and learning behavior, the teaching dimension includes teaching attitude, teaching method, and teaching ability, and the curriculum dimension includes course design and platform design, so as to establish a multiple linear regression equation. Taking the satisfaction with blended learning as the dependent variable Y, a regression analysis is conducted by including X1 (satisfaction with learning attitude), X2 (satisfaction with learning behavior), X3 (satisfaction with teaching attitude), X4 (satisfaction with teaching method), X5 (satisfaction with teaching ability), X6 (satisfaction with course design), and X7 (satisfaction with platform design) in turn. Results are shown in Table 15. R2 is 0.605, which is significantly greater than 0, representing that the regression model is scientific. It also shows that the independent variables formulated in this study, including the learning dimension (learning attitude, learning behavior), teaching dimension (teaching attitude, teaching method, teaching ability), and curriculum dimension (course design, platform design), can reflect 62.5% of the degree of change in the satisfaction of teaching. The *F*-value of the regression model is 140.206, and the corresponding significance level is 0.000 < 0.001, indicating a significant linear relationship between the predictor variables (learning attitudes, learning behaviors, teaching attitudes, teaching methods, teaching abilities, course design, and platform design) and the dependent variable satisfaction with blended learning. From the data in the table, the sig value corresponding to the t-statistic of any constant X is less than 0.05, in which all passed the t-test at the significance level of 0.05, indicating that this linear regression model is meaningful.

**Table 15:** Regression analysis of factors on the satisfaction with blended learning (beta value).

	Satisfaction with blended learning
**Learning dimensions**	
Learning attitude	0.221^***^
Learning behavior	0.125^**^
**Teaching dimensions**	
Teaching attitude	0.165^***^
Teaching method	0.181^***^
Teaching ability	0.116^*^
**Curriculum dimensions**	
Course design	0.192^***^
Platform design	0.096^*^
B	2.450
F	140.206^***^
Adjusted R2	0.605

^*^0.01 < sig < 0.05; ^**^0.001 < sig ≤ 0.01; ^***^sig ≤ 0.001.

From the Beta values, the regression coefficient of X1 (satisfaction with learning attitude) is 0.221; the regression coefficient of X2 (satisfaction with learning behavior) is 0.125; the regression coefficient of X3 (satisfaction with teaching attitude) is 0.165; the regression coefficient of X4 (satisfaction with teaching method) is 0.181; the regression coefficient of X5 (satisfaction with teaching ability) is 0.116; the regression coefficient of X6 (satisfaction with course design) is 0.192; the regression coefficient of X7 (satisfaction with platform design) is 0.096. That is, if the students’ satisfaction index of learning attitude, learning behavior, teaching attitude, teaching method, teaching ability, course design, and platform design all increase by 1 standard unit, the satisfaction index of blended learning will increase by 0.221, 0.125, 0.165, 0.181, 0.116, 0.192, and 0.096 basic units, respectively. Thus, learning attitudes, course design, and teaching methods are the factors that have the largest influence on the satisfaction of blended learning, whereas platform design has the least influence.

According to the results of the regression analysis in Table 15, the following regression equation was derived between the variables:

Satisfaction with Blended Learning = 2.450 + 0.221 * Learning Attitude + 0.125 * Learning Behavior + 0.165 * Teaching Attitude + 0.181 * Teaching Method + 0.116 * Teaching Ability + 0.192 * Course Design + 0.096 * Platform Design.

## Conclusion

College students are relatively satisfied with blended learning, and the satisfaction level of each dimension in learning is likewise in the upper-middle range, indicating that the majority of students are in favor of blended learning. According to the data, students are most satisfied with the teacher, followed by the course, and least satisfied with themselves. Specifically, students’ satisfaction with teachers’ teaching attitude, online learning platform, and self-learning awareness is high and at a good level. However, there are issues like students’ low interest in learning, lack of learning action, and failure to meet students’ needs in terms of teaching methods, teaching content resources, and learning platform technology. To support the development of blended learning and boost teaching effectiveness, further exploration and problem-solving are necessary. In some key statistics, there are notable differences in the satisfaction with blended learning. Specifically, there are significant differences in satisfaction ratings among different online resources, different online teaching forms, and different offline teaching methods. In terms of individual characteristics like grades, majors, and learning platforms, there is no significant difference in the satisfaction rating. For the influencing factors of college students’ satisfaction with blended learning, the results of correlation analysis show that the learning dimension has the strongest correlation with student’s satisfaction, followed by the curriculum dimension, and the teaching dimension has the weakest correlation. Thus, further regression analysis reveals significant positive correlations between student’s satisfaction with blended learning and learning attitude, learning behavior, teaching attitude, teaching method, teaching ability, curriculum design, and platform design. Among them, learning attitudes, course design, and teaching methods have a greater influence on students’ satisfaction with blended learning, and platform design has the least influence.

In the comparative analysis, there is a significant difference in students’ satisfaction with the blended learning of the Civics and Political Science class based on different offline teaching methods. The mutual discussion and communication style, as well as the student-led teaching style, are more popular among students because these two teaching methods emphasize the students’ initiative and allow students to fully interact with their classmates and teachers, and it also enable the teacher to better tailor their teaching to the students’ actual situation. Therefore, it is advocated to put interactive communication at the core of classroom teaching. The online teaching process can attract students’ attention and increase their interest in learning through questions, answers, discussions, and game-based activities. Offline teaching is problem-oriented, integrating communication and discussion into classroom teaching as a necessary part, increasing students’ participation, and focusing on cultivating students’ ability to discern, think, and express. Based on different online teaching forms, there were significant differences in students’ satisfaction with blended learning in the Civics courses. The mean value of student satisfaction with blended learning is highest in the online teaching form with a fixed location and fixed time. Due to integrity issues with students’ online learning and their irresponsible behavior toward learning, the original purpose of online learning is being compromised. Therefore, online learning should strengthen its supervision of students’ learning. This can be achieved by giving students some degree of “freedom” while continually monitoring their selection of the online learning mode, time, and location, and guiding them to make organized arrangements for their own learning that fall within a reasonable range. Additionally, we should teach students self-awareness of online learning, independent learning behaviors, and the ability to promptly adjust their learning progress in response to the course dynamics.

The results of Pearson correlation analysis show that the learning dimension has the strongest correlation with satisfaction and is the primary factor influencing students’ satisfaction with blended learning. The findings of the regression analysis further support the idea that students’ satisfaction with blended learning is significantly influenced by their learning behavior and learning attitude in the learning dimensions. Learning behaviors and learning attitudes mainly comprise important factors such as learning interest, learning awareness, learning self-efficacy, learning concentration, learning reflection, and the level of communication and interaction, which together affect students’ satisfaction with blended learning in universities. Our results are in alignment with the literature reporting that achievement in blended courses is influenced to a greater extent by students’ conceptions of learning, their ability to accept responsibility for their learning, and the degree of interactivity outside of the classroom (Mitchell and Honore, 2007; Moore and Gilmartin, 2010; Bliuc et al., 2011; Chou and Chou, 2011; Smyth et al., 2012). In comparison with the average satisfaction of teachers, the average satisfaction of teachers’ teaching attitudes is greater than the average satisfaction of teaching methods and teaching abilities, and the impact of teachers’ teaching methods is greater, these factors include teachers’ positive or negative attitudes toward technology use, learners’ proficiency levels, teachers’ training, teachers’ and students’ accessibility to technology, and cost. Each one of these factors plays a vital role in decisions regarding implementing a blended learning approach in language classrooms (Sharma and Barrett, 2008). Students have the lowest mean value of satisfaction with themselves when compared to the mean value of satisfaction with teachers and the mean value of satisfaction with courses. The student’s actual performance in class, which serves as the focus of educational activities, has an important influence on their satisfaction. Only when the student’s interest in learning is completely mobilized can a positive teaching effect be realized. In response to the issues of students’ low self-satisfaction, lack of learning interest and learning motivation in blended learning, it is necessary not only to impart relevant knowledge during actual teaching but also to foster the students’ enjoyment in learning knowledge on the basis of it.

## Data availability statement

The raw data supporting the conclusions of this article will be made available by the authors, without undue reservation.

## Author contributions

XC wrote the original manuscript. XC and WM performed the data collection and treatment. YD reviewed and revised the manuscript. All authors contributed to the article and approved the submitted version.

## Funding

This research was supported by Chengdu University of Technology “Double First-Class” initiative Construction Philosophy and Social Sciences Key Construction Project, No: ZDJS202306, Special Project of “Research on Informatization of Higher Education” of the Chinese Society of Higher Education, and Sichuan Province Chengdu University of Technology 2022 Talent Cultivation Quality and Teaching reform project.

## Conflict of interest

The authors declare that the research was conducted in the absence of any commercial or financial relationships that could be construed as a potential conflict of interest.

## Publisher’s note

All claims expressed in this article are solely those of the authors and do not necessarily represent those of their affiliated organizations, or those of the publisher, the editors and the reviewers. Any product that may be evaluated in this article, or claim that may be made by its manufacturer, is not guaranteed or endorsed by the publisher.
